# Recombinant Enterovirus A71 Subgenogroup C1 Strains, Germany, 2015 

**DOI:** 10.3201/eid2210.160357

**Published:** 2016-10

**Authors:** Sindy Böttcher, Patrick E. Obermeier, Katrin Neubauer, Sabine Diedrich, the Laboratory Network for Enterovirus Diagnostics

**Affiliations:** Robert Koch Institute, Berlin, Germany

**Keywords:** enterovirus, enterovirus A71, meningitis, picornavirus, surveillance, acute flaccid paralysis, Germany, viruses

**To the Editor:** Enterovirus A71 (EV-A71) strains circulate worldwide, and numerous outbreaks have been reported from Asia, Australia, Europe, and America (*1*). Symptomatic infections range from mild febrile illness or characteristic diseases such as hand, foot and mouth disease to severe neurologic disorders such as meningitis/encephalitis and acute flaccid paralysis. EV-A71 infections are usually asymptomatic and self-limiting but can also result in life-threatening complications such as pulmonary edema and cause death, predominantly in children <5 years of age. On the basis of viral protein 1 (VP1) sequences, 3 genogroups (A, B, C), including different subgenogroups (B0–B5, C1–C5), have been defined (*2*,*3*). Additional genogroups (D, E, F, G) have been proposed (*4*,*5*). In Europe, C1 and C2 strains have circulated predominantly within the past 2 decades, and recent introduction of C4 strains has been reported (*6*,*7*). Within subgenogroup C1, a lineage is replaced by the subsequent lineage over time (*8*). 

National enterovirus surveillance (EVSurv) in Germany monitors polio-free status by testing fecal or cerebrospinal fluid (CSF) samples from hospitalized patients with suspected meningitis/encephalitis or acute flaccid paralysis. Enterovirus typing, using molecular and virologic methods, is performed within a laboratory network for enterovirus diagnostics. Since 2006, ≈2,500 samples have been tested annually; 25%–30% were enterovirus positive. Of the typed strains, 0.8%–12.7% were identified as EV-A71 (2006, 0.8%; 2007, 6.8%; 2008, 0.9%; 2009, 3.4%; 2010, 12.7%; 2011, 2.3%; 2012, 2.8%; 2013, 8.6%; 2014, 2.7%), indicating peaks with increased EV-A71 detection rates. Molecular characterization based on the VP1 region of a subset of EV-A71–positive samples revealed that C2 was the predominant subgenogroup in Germany from 2006 to 2014. Subgenogroups B5, C1, and C4 have also been identified, but less frequently (Technical Appendix Table 3). 

In 2015, a total of 419 samples tested enterovirus positive within EVSurv. Of these, 43 fecal specimens and 1 CSF specimen tested EV-A71 positive (11.2% of the typed enteroviruses); these samples were obtained from patients with signs of meningitis/encephalitis hospitalized in 25 secondary and tertiary care hospitals from 13 of 16 federal states of Germany. Thirty-six strains were further characterized at the National Reference Centre for Poliomyelitis and Enteroviruses (Technical Appendix Table 2). Seventeen strains were identified as C2 by using the RIVM Enterovirus Genotyping Tool Version 1.0 (http://www.rivm.nl/mpf/enterovirus/typingtool) based on the VP1 region sequences (*9*). Sequence analyses of the remaining 19 strains revealed highest nucleotide identity (90%–93%) with recently circulating C1 strains from GenBank. Phylogenetic analysis that used the neighbor-joining tree algorithm showed separate clustering of these strains within the C1 subgenogroup (Figure). In contrast to the VP1 tree, phylogenetic analyses based on the 5′ untranslated region (UTR) and the P2 and P3 regions revealed different clustering of the German 2015 EV-A71 C1-like group (Technical Appendix Figure). In line with these phylogenetic tree topologies, we found highest nucleotide identities to subgenogroup B3 and C2-like strains for 5′ UTR (both 90%), whereas P2 and P3 regions showed highest nucleotide identity of 82% and 84%, respectively, with C4 strains identified in China. We found no specific amino acid changes within the conserved major antigenic sites of the capsid proteins. However, we observed a V16M change in VP1. Few EV-A71 strains (all B1) also carry a methionine at this position, including the outbreak strains from Bulgaria (1975) and Hungary (1978). Also, we identified a valine residue at position 262 in VP1. Tee et al. have proposed that toggling of amino acids isoleucine and valine at this position in recent C1 lineages generates antigenic novelty (*8*). Within the 5′ UTR, a C526U change has been proposed to affect replication efficiency (*10*). All isolates belonging to the new EV-A71 C1 variant carried uracil at this position. In addition, we found a 2-nucleotide deletion within the spacer 2 region between the internal ribosome entry site and the coding region, similar to the EV-A71 prototype BrCr and the C2-like strains (GenBank accession nos. HM622392, HM622391, JQ280307) (data available on request).

**Figure:**
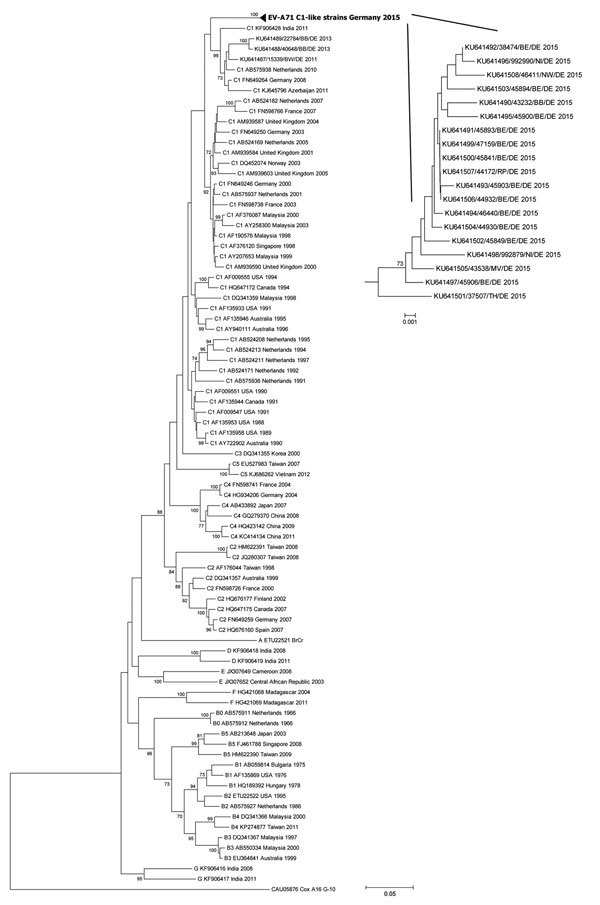
Phylogenetic tree based on complete viral protein 1 (VP1) nucleotide sequences of the strains identified within the German enterovirus surveillance (**bold**) and a representative set of enterovirus A71 strains available from GenBank (891 bases, corresponding to nucleotide positions 2439–3329 in the prototype BrCr ETU22521). The tree was constructed by using the neighbor-joining method (Kimura 2-parameter model) with 1,000 replicates through MEGA 6.06 (http://www.megasoftware.net/). Coxsackievirus A16 prototype (CAU05876) was used as the outgroup. Only bootstrap values >70 are shown. Genogroup and subgenogroup assignment, GenBank accession number, country and year of isolation are provided in the virus names. The enlarged subtree includes enterovirus A71 C1-like strains detected in Germany in 2015. Virus names contain strain number, abbreviation of federal state, country, and year of isolation. BE, Berlin; BB, Brandenburg; DE, Germany; MV, Mecklenburg Western Pomerania; NI, Lower Saxony; NW, Northrhine-Westphalia; RP, Rhineland Palatinate; TH, Thuringia. Scale bars indicate nucleotide substitution per site.

Our findings highlight the need for molecular surveillance of enteroviruses to identify new variant strains with potential for increased virulence and pathogenicity. One limitation of the EVSurv is the lack of detailed clinical data because the request form deliberately asks for only basic cardinal symptoms justifying the clinical suspicion of meningitis/encephalitis or acute flaccid paralysis. Nevertheless, all patients had been hospitalized, suggesting severe disease. Besides characteristic symptoms (including nuchal rigidity, headache, fever, and vomiting), cerebral seizures, myoclonia, ataxia, petechiae, and stomatitis were also mentioned for some patients tested for the new variant C1-like strains described here. All patients, but 1, were <5 years of age (Technical Appendix Table 1). Therefore, pediatricians, in particular, should be aware of this new recombinant, potentially more pathogenic, strain and intensify diagnostic work-ups to better monitor EV-A71 circulation. In addition to CSF samples, fecal samples, throat swab specimens, and samples related to other clinical prodromes (e.g., vesicle fluids in cases of hand, foot and mouth disease) should be obtained. Particular attention should be paid to measures in daycare centers to prevent large outbreaks of enterovirus-associated meningitis/encephalitis.

Technical Appendix Clinical specimens and methods used in study of recombinant enterovirus A71 subgenogroup C1 strains, Germany, 2015.
