# Supplementary material for: Recombinant Enterovirus A71 Subgenogroup C1 Strains, Germany, 2015
Source: Emerg Infect Dis. 2016 Oct;22(10):1843–6. doi: 10.3201/eid2210.160357 (PMC5038430; doi:10.3201/eid2210.160357)
Supplement: Technical Appendix — Clinical specimens and methods used in study of recombinant enterovirus A71 subgenogroup C1 strains, Germany, 2015. [file 16-0357-Techapp-s1.pdf]

Article DOI: http://dx.doi.org/10.3201/eid2210.160357

Recombinant Enterovirus A71 Subgenogroup C1 Strains, Germany, 2015

Technical Appendix

Clinical Specimens

All samples were collected from hospitalized patients with symptoms of meningitis/encephalitis within the German enterovirus surveillance network. Specimens were sent to a network laboratory that was participating in the Laboratory Network for Enterovirus Diagnostics (LaNED), including the National Reference Centre for Poliomyelitis and Enteroviruses (Berlin, Germany). All patient data were analyzed pseudonymously.

RNA was isolated directly from fecal samples or from cell culture supernatant by using the QIAGEN RNA Viral Mini Kit (QIAGEN, Hilden, Germany). Amplification was performed for all PCRs by using the One-Step-RT-PCR Kit (QIAGEN), followed by nested PCR using HotStarTaq-Mastermix (QIAGEN), according to the manufacturer's protocol. Reverse transcription PCR (RT-PCR) and nested PCR was done with 600 nM of forward and reverse primers as mentioned in Technical Appendix Table 1. The temperature profile for the VP1 region was the following: 10 min 22°C, 45 min 45°C, 15 min 95°C for RT, followed by 40 cycles of 30 s 94°C, 30 s 46°C, 90 s 72°C, and final elongation for 10 min at 72°C. The second PCR was carried out by using a touchdown protocol with 10 cycles of 30 s 94°C, 30 s 60°C, 90 s 72°C, with a decrease of 1°C of the annealing temperature per cycle, followed by 30 cycles of 30 s 94°C, 30 s 50°C, 90 s 72°C, and final elongation for 10 min at 72°C. Amplification of the 5′ untranslated region (UTR) was done as described (*1*). Temperature profile for all other PCRs was as follows: 30 min 50°C, 15 min 95°C for RT, followed by 35 cycles of 30 s 94°C, 30 s 50°C, 90 s 72°C, and final elongation 10 min at 72°C. Nested PCR was done in 35 cycles of 30 s 94°C, 30 s 50°C, 90 s 72°C, followed by final elongation of 10 min 72°C. The resulting products were sequenced with primers used for amplification (BigDye 3.1 kit, Applied Biosystems,

Weiterstadt, Germany). PCR products of VP3 and 3ABC PCR were extracted from the agarose gel using QIAGEN Mini Elute Gel Kit (QIAGEN), according to the manufacturer's protocol.

Sequences were assembled with Sequencher software version 5.2.4 (https://www.genecodes.com/). Alignments were performed by using MAFFT (*2*). Phylogenetic relationships among strains circulating in Germany and representative strains taken from GenBank were estimated with the neighbor joining method based on the Kimura 2-parameter model conducted in MEGA6 by using a bootstrap procedure with 1,000 replicates (*3*).

References

1. Kuryk L, Wieczorek M, Diedrich S, Böttcher S, Witek A, Litwinksa B. Genetic analysis of poliovirus strains isolated from sewage in Poland. J Med Virol. 2014;86:1243–8. PubMed http://dx.doi.org/10.1002/jmv.23803

2. Katoh K, Standley DM. MAFFT multiple sequence alignment software version 7: improvements in performance and usability. Mol Biol Evol. 2013;30:772–80 and. PubMed http://dx.doi.org/10.1093/molbev/mst010

3. Tamura K, Stecher G, Peterson D, Filipski A, Kumar S. MEGA6: Molecular Evolutionary Genetics Analysis version 6.0. Mol Biol Evol. 2013;30:2725–9 and. PubMed http://dx.doi.org/10.1093/molbev/mst197

4. Leitch EC, Harvala H, Robertson I, Ubillos I, Templeton K, Simmonds P. Direct identification of human enterovirus serotypes in cerebrospinal fluid by amplification and sequencing of the VP1 region. J Clin Virol. 2009;44:119–24. PubMed http://dx.doi.org/10.1016/j.jcv.2008.11.015

.

Technical Appendix Table 1. Overview and accession numbers of enterovirus A71 strains described in this study

Strain	GenBank accession no.	Collection date	Federal state	Age, y	Sex	Diagnosis
45841/BE/DE 2015	KU641500	2015 Jun	Berlin	Newborn	F	Meningitis
45893/BE/DE 2015	KU641491	2015 Jul	Berlin	4 mo	M	Meningitis
38474/BE/DE 2015	KU641492	2015 Jul	Berlin	2	F	Meningitis
45903/BE/DE 2015	KU641493	2015 Jul	Berlin	2	F	Meningitis
46440/BE/DE 2015	KU641494	2015 Jul	Berlin	3	F	Meningitis
45900/BE/DE 2015	KU641495	201 Jul	Berlin	3	F	Meningitis
43232/BB/DE 2015	KU641490	2015 Jul	Brandenburg	3	F	Meningitis
45849/BE/DE 2015	KU641502	2015 Jul	Berlin	2	F	Meningitis
45894/BE/DE 2015	KU641503	2015 Jul	Berlin	3	M	Meningitis
992990/NI/DE 2015	KU641496	2015 Aug	Lower Saxony	4	F	Meningitis
37507/TH/DE 2015	KU641501	2015 Aug	Thuringia	4	M	Meningitis
44930/BE/DE 2015	KU641504	2015 Aug	Berlin	11 mo	F	Meningitis
43538/MV/DE 2015	KU641505	2015 Aug	Mecklenburg Western Pomerania	3	M	Meningitis
44932/BE/DE 2015	KU641506	2015 Aug	Berlin	1	M	Meningitis
44172/RP/DE 2015	KU641507	2015 Sep	Rhineland Palatinate	1	F	Meningitis
45906/BE/DE 2015	KU641497	2015 Sep	Berlin	17	M	Meningitis
992879/NI/DE 2015	KU641498	2015 Oct	Lower Saxony	2 mo	F	Meningitis
46411/NW/DE 2015	KU641508	2015 Oct	Northrhine-Westphalia	5	F	Meningitis
47159/BE/DE 2015	KU641499	2015 Nov	Berlin	1 mo	F	Meningitis

Technical Appendix Table 2. Primers used for amplification of enterovirus A71*

Primer	Region amplified	Sequence 5′–3′	Orientation	Location†	Reference
NRZ 1	5′UTR	CAA GCA CTT CTG TTT CCC CGG	Sense	167–187	(*1*)
NRZ 2	5′UTR	ATTGTCACCATAAGCAGCCA	Antisense	602–585	(*1*)
NRZ 5	5′UTR	TAC TTC GAG AAR CCY AGT A	Sense	248–266	(*1*)
NRZ80	5′UTR	AAC ACG GAC ACC CAA AGT A	Antisense	565–547	(*1*)
NRZ 338	VP4	TGG CGG CCT GCC YAT GG	Sense	369–385	This study
NRZ 339	VP4	TCC TCC GGC CCC TGA ATG C	Sense	448–466	This study
NRZ 340	VP4	TGT GAT ATA GGR ATc CCA GCA TCR AG	Antisense	1471–1446	This study
NRZ 341	VP3	AGY AAR TTC CAY CAA GGR GCR C	Sense	1293–1314	This study
NRZ 342	VP3	TCR ATC ATR CTC TCR TCA CTA G	Antisense	2635–2611	This study
NRZ 343	VP3	GCY CCA ATY TCA GCR GCT TG	Antisense	2599–2580	This study
NRZ 360	VP3 seq	GCT GGA GCT GTG TCA GGT GG	Sense	1841–1860	This study
NRZ 361	VP3 seq	TGC GTG CCC AGC ATA GCG	Antisense	2173–2156	This study
NRZ 131	VP1	CCN TGG ATH AGY AAC ACN CAY T	Sense	2220–2241	(*4*)
NRZ 209	VP1	CTR ACY GGR TAR TGY TTY CT	Antisense	3553–3534	This study
NRZ 210	VP1	THT GGT AYC ARA CAA AYT WYG TNG THC C	Sense	2299–2326	This study
NRZ 211	VP1	CCM ACR TAD ATD GCN CCN GAY TGY TGN CC	Antisense	3367–3339	This study
NRZ 337	VP1 seq	ACG TTC GGT GAG CAC AAG C	Sense	3066–3084	This study
NRZ 344	2ABC	ACR TTC GGT GAR CAC AAG CAG	Sense	3066–3086	This study
NRZ 345	2ABC	TGG TGA TYA GGA TTT ACA TGA GGA TG	Sense	3178–3203	This study
NRZ 346	2ABC	GGT GTT TGC TCT TGA ACT GCA T	Antisense	4431–4410	This study
NRZ 353	2ABC seq	GGC CAG TGA GTA TTA CCC GG	Sense	3584–3603	This study
NRZ 347	3ABC	TCC AAC CTT GAG CAG TCY GC	Sense	4257–4276	This study
NRZ 348	3ABC	ATG GAA TGT TTC ATA GAG GTG CC	Antisense	6533–6511	This study
NRZ 349	3ABC	GCC ATT CTA AGG TAA ACT GAG TC	Antisense	6505–6483	This study
NRZ 362	3ABC seq	CGG ATC TTG GCC GAT TGG A	Sense	4843–4861	This study
NRZ 363	3ABC seq	TGC CAC CAA TGT GAA TGC C	Antisense	5880–5862	This study
NRZ 350	3D	TGA GCA TRG ADG ADG CYT G	Sense	6220–6237	This study
NRZ 351	3D	ATG AAR TTY TAY ATG GAY AAG TAT GG	Sense	6357–6382	This study
NRZ 352	3D	AGA TTH CTG GTG GGG TTS AGB T	Antisense	7360–7339	This study
NRZ 370	3D seq	ATG AYT CAG TGT ACY TSA GRA TGR C	Sense	6481–6505	This study
NRZ 371	3D seq	GCT GAW CCR GTK AYY GTM CCW GG	Antisense	6562–6540	This study

*UTR, untranslated region; VP, viral protein; seq, used for sequencing only.
†Location according to EV-A71 prototype strain BrCr (ETU25521).

Technical Appendix Table 3. Number of samples analyzed in the German enterovirus surveillance and enterovirus A71 genotyping results, 2006–2015*

Samples	2006	2007	2008	2009	2010	2011	2012	2013	2014	2015
Total no. samples tested	1,856	2,135	3,207	2,526	2,804	2,992	2,859	3,413	2,461	2,158
No. EV positive	645	657	1,139	501	772	740	670	1,239	506	419
No. EV typed	358	370	928	383	575	649	581	1,118	482	392
EV-A71 positive	3	25	8	13	73	15	16	96	13	44
EV-A71/EV typed, %	0.8	6.8	0.9	3.4	12.7	2.3	2.8	8.6	2.7	11.2
Subgenogroup										
B5			2					1		
C1					1	1	1	4	1	19
C2	2	13	1	3	52	7	8	58	7	17
C4						5	3	12		
ND	1	12	5	10	20	2	4	21	5	8

*EV, enterovirus; ND, not determined.

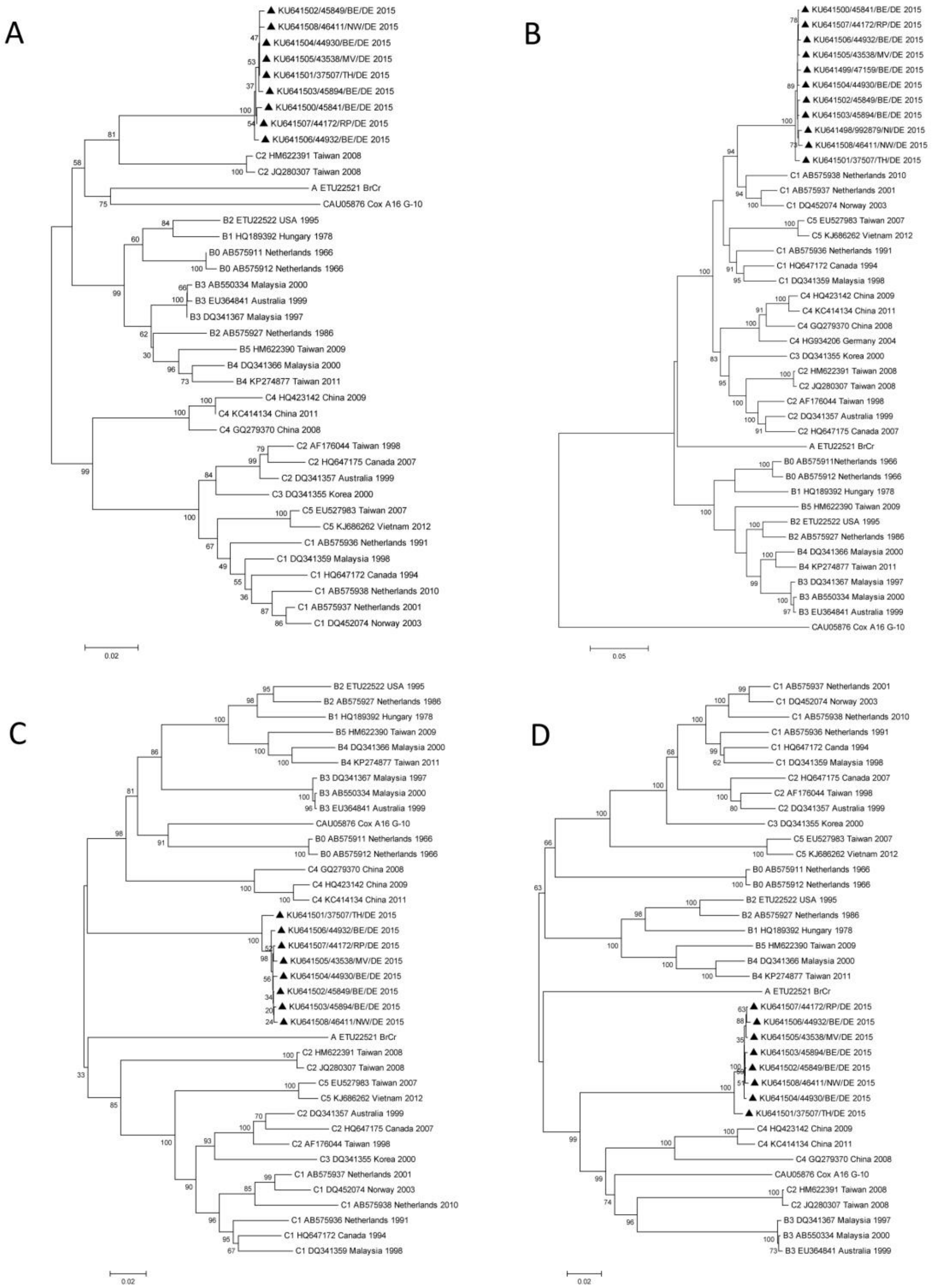

Technical Appendix Figure. Phylogenetic tree based on complete 5′untranslated region (A, 562 ntd), P1 (B, 2,586 nt), P2 (C, 1,734 nt), and P3 (D, 2,262 nt) region of the strains identified within the German enterovirus surveillance and a representative set of enterovirus A71 available from GenBank. Trees were

constructed by using the neighbor-joining method (Kimura 2-parameter model) with 1,000 replicates through MEGA 6.06 (http://www.megasoftware.net/). Coxsackievirus A16 prototype (CAU05876) was used as the outgroup. Only bootstrap values >70 are shown. Scale bar represents nucleotide substitutions per site. Genogroup and subgenogroup assignment, GenBank accession number, country, and year of isolation are provided in the virus names. Virus names of the German strains contain strain number, abbreviation of federal state (BB, Brandenburg; BE, Berlin; MV, Mecklenburg Western Pomerania; NI, Lower Saxony; NW, Northrhine-Westphalia; RP, Rhineland Palatinate; TH, Thuringia), country (DE, Germany), and year of isolation.
